# Discrimination, Coping, and DNAm Accelerated Aging Among African American Mothers of the InterGEN Study

**DOI:** 10.3390/epigenomes9020014

**Published:** 2025-05-04

**Authors:** Alexandria Nyembwe, Yihong Zhao, Billy A. Caceres, Daniel W. Belsky, Calen Patrick Ryan, Brittany Taylor, Morgan T. Morrison, Laura Prescott, Stephanie Potts-Thompson, Arezo Aziz, Fisola Aruleba, Erica Matute-Arcos, Olajide Williams, Cindy Crusto, Jacquelyn Y. Taylor

**Affiliations:** 1 Sue & Bill Gross School of Nursing, University of California, Irvine, 854 Health Sciences Rd., Irvine, CA 92697, USA; 2 Center for Research on People of Color, School of Nursing, Columbia University, 560 W 168th St, New York, NY 10032, USA; yz2135@cumc.columbia.edu (Y.Z.); bac2134@cumc.columbia.edu (B.A.C.); mtm2206@cumc.columbia.edu (M.T.M.); lp2880@cumc.columbia.edu (L.P.); sp3879@cumc.columbia.edu (S.P.-T.); aa446@cumc.columbia.edu (A.A.); em3990@cumc.columbia.edu (E.M.-A.); jyt2116@cumc.columbia.edu (J.Y.T.); 3 Robert N. Butler Columbia Aging Center, Columbia University, 722 West 168th Street, New York, NY 10032, USA; db3275@cumc.columbia.edu (D.W.B.); cpr2139@cumc.columbia.edu (C.P.R.); 4 School of Nursing, University of Pittsburgh, 3500 Victoria Street, Victoria Building, Pittsburgh, PA 15213, USA; brt148@pitt.edu; 5 School of Medicine, The City College of New York (CUNY), Harris Hall, 160 Convent Avenue, New York, NY 10031, USA; oaruleb001@citymail.cuny.edu; 6 Vagelos College of Physicians and Surgeons, Columbia University, 630 W 168th St, New York, NY 10032, USA; ow11@cumc.columbia.edu; 7 Keck School of Medicine, University of Southern California (USC), 1975 Zonal Ave, Los Angeles, CA 90033, USA; crusto@usc.edu

**Keywords:** racial discrimination, social support, accelerated aging, epigenetics, African American women

## Abstract

**Background:** Racial discrimination experiences are associated with the activation of stress biology pathways and signs of accelerated biological aging, including alterations in DNA methylation (DNAm). Coping strategies may mitigate stress from racial discrimination and protect against long-term adverse health outcomes. **Methods:** We conducted a secondary analysis of data from the Intergenerational Impact of Genetic and Psychological Factors on Blood Pressure cohort, an all-African-American sample, to test the hypothesis that social support can protect against accelerated biological aging associated with experiences of racial discrimination. We measured biological aging from saliva DNAm using six epigenetic clocks. Clock values were residualized on participant age and the estimated proportion of epithelial cells contributing to the DNA sample and standardized to M = 0, SD = 1 within the analysis sample. The primary analysis was focused on the second-generation PhenoAge and GrimAge clocks and the third-generation DunedinPACE “speedometer,” which previous studies have linked with racial discrimination. **Results:** In our sample (*n* = 234; mean age = 31.9 years; SD = 5.80), we found evidence consistent with our hypothesis in the case of the PhenoAge clock, but not the other clocks. Among mothers who did not seek social support, experiences of racial discrimination were associated with an older PhenoAge (b = 0.26, 95% CI = 0.02–0.50, *p* = 0.03). However, social-support seeking mitigated this risk; at the highest levels of social support, no adverse consequences of discrimination were observed (interaction b = −0.01, 95% CI = −0.02–−0.00, *p* = 0.03). **Conclusions:** The replication of results is needed. Future research should also investigate additional adaptive and maladaptive coping strategies utilized by African American women and mothers to identify protective measures that influence health outcomes.

## 1. Introduction

Racism is a public health crisis [1]; it manifests at both the individual and institutional levels and stems from an ideology of superiority that classifies and ranks different racial groups while fostering stereotypes and differential treatment of those perceived as inferior [2]. Although explicit support for racist attitudes has declined over time, racism continues to operate through institutional structures and implicit biases [1,3]. Research indicates that racism contributes to negative attitudes and experiences of discrimination among racial and ethnic minorities [4]. Racial discrimination, which includes negative attitudes and beliefs about racial minorities, remains prevalent in employment, housing, healthcare, and criminal justice sectors, despite protective laws [5]. These discriminatory practices reinforce harmful stereotypes and can produce damaging effects—whether experienced overtly or subtly—at both the individual and institutional levels.

Racial discrimination often manifests in daily experiences, such as unfair treatment by police or while shopping in stores. Such encounters can have profound impacts on individuals’ wellbeing. In a University of Rochester Medical Center study, more than three-quarters (82.6%) of African Americans reported that experiences of racial discrimination interfered with productivity and “made life much harder” [6]. Carter and Forsyth examined emotional and psychological reactions to experiences with racism and the help seeking strategies used to deal with those reactions among racial and ethnic minority adults [7]. They found that adults who reported direct experiences of racial discrimination exhibited heightened levels of race-related anxiety, guilt, shame, avoidance, and hypervigilance compared to adults who did not report such experiences. These experiences are especially concerning for African American women, who have reported chronic discrimination experiences at the intersection of race and gender, termed gendered racism [8], which can result in distinct psychosocial stress [9,10] and lead to poor health outcomes among this subgroup [11,12,13].

### 1.1. Racial Discrimination as a Stressor

Experiencing racial discrimination may trigger a powerful and complex stress response. Stress typically involves an environmental stimulus (real or perceived), a biological response, and a physiological response [14]. The stress response involves a sequence of brain activations, eventually activating systems such as the hypothalamic–pituitary–adrenal axis [14,15] and the cardiovascular system [16]. The prolonged or repeated activation of these systems can impair organ function and increase the risk of disease and mortality [15,16,17]. The concept of allostatic load, which describes the cumulative wear and tear caused by prolonged activation of stress response systems [14,18], is another biological mechanism which explains the relationship between stress and physical health and has been reportedly higher in African American women compared to that in White women [19]. These systemic responses suggest that psychosocial stressors, when persistent, may biologically embed themselves over time. Prior studies indicate that psychosocial stress resulting from experiences with racial discrimination is associated with epigenetic aging [20], reinforcing the hypothesis that racial discrimination can function as a stressor that also contributes to accelerated biological aging [20,21,22,23,24,25].

Racial discrimination functions as both an acute and chronic stressor with biological consequences that may be partially explained through the progressive dysregulation of stress response systems [21,24,26,27]. African American women often cite individual-level stressors in multiple environments including the workplace [10], healthcare [28,29], and stress experienced by those within their social networks, including family members and friends [28]. Such stressors may compound other existing health burdens, which may then significantly affect overall health and well-being across their lifetime [30,31,32].

### 1.2. Coping with Stress

Coping strategies refer to how individuals respond to a stressor [33,34] and vary widely among individuals. These strategies may serve as protective barriers against discrimination experiences and determine whether a physiological or psychological stress response occurs [33,35]. Adaptive coping strategies involve intentional behavioral efforts to manage stress effectively and are believed to protect against adverse stress responses [36]. Such coping mechanisms among African American women are diverse and include practices such as seeking social support, engaging in religious activities, and cultivating resilience [10,37]. Social support encompasses the resources and sense of connection an individual derives from personal relationships and their broader social network [38]. Such support can be functional (perceived or tangible assistance) or structural (marital status and the frequency of social interaction); both forms are widely recognized as protective factors that can help mitigate stress responses through various physiological pathways [15]. While African American women often seek support from relatives, friendships, and significant others or spouses to manage general and race-related stressors [39,40], less is known about this coping strategy’s association with biological aging and whether it moderates any association between racial discrimination and biological aging among this subgroup.

Experiences of racial discrimination may become biologically embedded through stress-induced epigenetic modifications, particularly DNA methylation (DNAm). Additionally, the chronic or repeated activation of the stress-response system may alter the regulation of some genes involved in cellular aging. Our hypothesis positions social support as a potential buffer in this pathway, which may mitigate the impact of racial discrimination on DNAm age acceleration. This study builds on prior research suggesting that social support moderates the association between racial discrimination and blood pressure in African American women [41] and extends this framework to the domain of epigenetic aging.

### 1.3. Biological Aging

Epigenetics describes changes in the regulation of gene expression within the body throughout the lifespan and involves chemical modifications that may be influenced by environmental factors and personal behaviors or experiences, including psychosocial exposures such as stress [42]. DNAm is one such mechanism, which involves the addition or removal of a methyl molecule to DNA bases, blocking transcription factors from binding to the sequence and interfering with gene expression [43]. Chronic stress stemming from racial discrimination experiences has been associated with alterations in DNAm patterns [20,44,45], which may drive downstream effects on biological aging [46,47,48]. In this study, we focused on DNAm-based measurements called “epigenetic clocks” [49,50].

Epigenetic clocks are DNAm algorithms that summarize the progress or the pace of biological aging. Researchers developed the first generation of epigenetic clocks by modeling chronological age differences between research participants [49,51]. Next, researchers developed a second generation of epigenetic clocks by modeling survival time differences between research participants [52,53]. Then, researchers developed a third generation of epigenetic clocks by modeling differences between research participants in their rate of multi-organ-system deterioration (termed “Pace of Aging”) [54,55]. Second- and third-generation clocks are robust predictors of morbidity and mortality [56,57]. They also show consistent evidence of association with several social determinants of health, in particular, markers of poverty and socioeconomic status, and tend to indicate an older biological age and a faster pace of aging in African Americans as compared with those in White Americans [21,58]. However, the mediators of these associations between social determinants of health and epigenetic clock measures of biological aging are not well-characterized.

Several studies have explored associations between psychosocial factors and epigenetic clocks, although findings vary, with minimal African American representation in the studies’ samples used to examine this potential association [20,59]. Zannas and colleagues found that cumulative lifetime stress, primarily personal life stressors, predicted accelerated aging in a sample of 392 African American adults [60]. Similarly, Brody and colleagues examined racial discrimination and cellular aging between these two samples (low- vs. high-support family environments) of African American youth in rural Georgia, United States [61]. They found that higher levels of racial discrimination predicted accelerated epigenetic aging in youth from less supportive environments [61]. Previously, we found that seeking social support may moderate the association between racial discrimination and blood pressure in young African American mothers [41]. To our knowledge, there are no studies that have investigated the moderating effects of seeking social support coping on the association between racial discrimination experiences and DNAm age acceleration or the pace of aging among young African American mothers. The present study aims to explore this association; we hypothesized that racial discrimination experiences would be associated with older biological age and a faster pace of aging, and that this association would be attenuated by adaptive-coping-strategy-seeking social support.

## 2. Results

The sample characteristics are reported in Table 1. Our analysis sample consisted of *n* = 234 women (mean age = 31.9 years; SD = 5.80). The mean body mass index in the sample was 29.6 (SD = 8.13), and 22.6% (*n* = 53) reported current smoking. The mean systolic and diastolic blood pressure was 114 mmHg (SD = 13.5) and 73 mmHg (SD = 10.6), respectively. We assessed racial discrimination using the Experiences of Discrimination (EOD) Situation scale; participants reported a mean of 1.45 (SD = 1.93) racial discriminatory events in their lifetime. The average seeking social support coping score was 21.7 (SD = 7.2).

**Figure 1:**
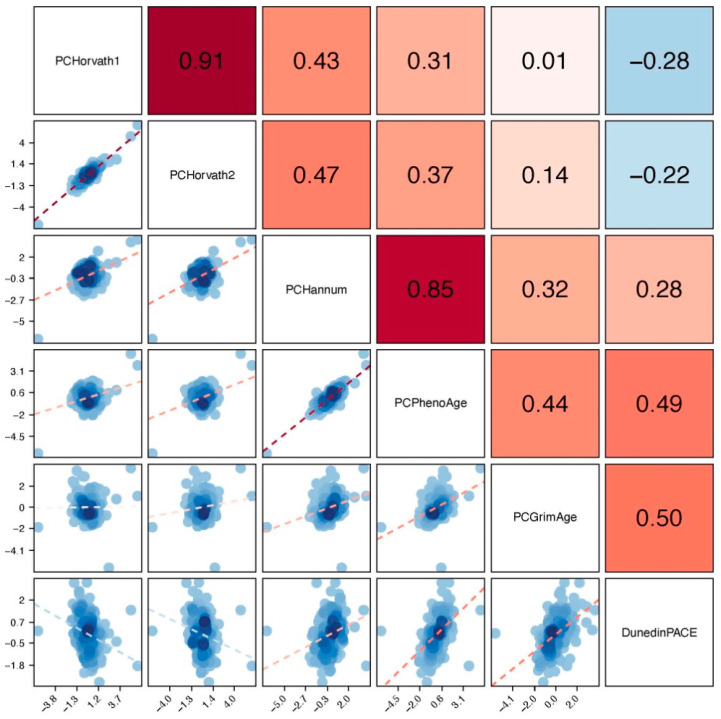
Correlation coefficient matrix between epigenetic clock estimates residualized on age and percent epithelial cells. The darker colors indicate stronger, positive correlations between epigenetic clocks; lighter colors indicate weaker, negative correlations.

**Figure 2:**
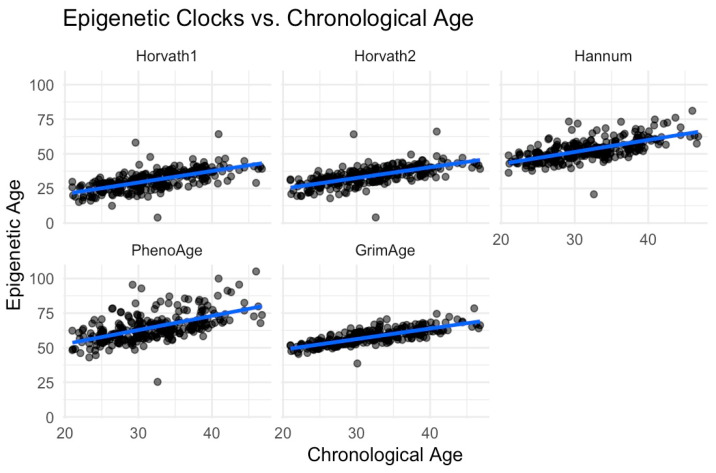
Epigenetic clocks vs. chronological age. Scatterplots display the association between chronological age and five DNA-methylation-based Horvath 1 (mean: 30.7, SD: 7.42), Horvath 2 (mean: 33.9, SD: 7.00), Hannum, PhenoAge (mean: 64.6, SD: 10.4), and GrimAge (mean: 57.6,SD: 5.68) epigenetic clocks. The blue lines represent the linear trend of the data.

**Figure 3:**
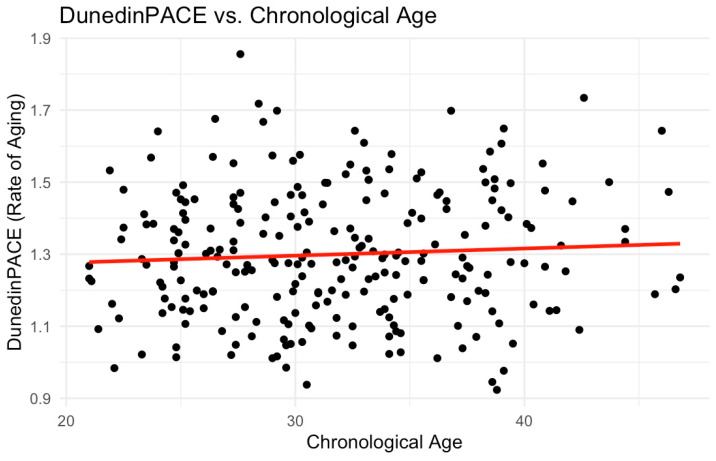
DunedinPACE vs. chronological age. This scatterplot displays the association between chronological age and the DunedinPACE epigenetic clock among this sample of young African American mothers, which estimates the pace of biological aging. Rates above 1.0 indicate faster aging, while rates below 1.0 indicate slower aging. The mean for this sample was 1.30 (SD: 0.18). The red line represents the linear trend of the data.

We computed epigenetic clocks from DNA methylation assayed from saliva samples using the Illumina EPIC array and published code pipelines [55,62]. Clock values were residualized on participant age, and the estimated proportion of epithelial cells contributing to the DNA sample and standardized to M = 0, SD = 1 within the analysis sample. We correlated clock values; the strongest correlations observed were between the Horvath Multi-Tissue (“Horvath1”) and Horvath Skin and Blood (“Horvath2”) clocks and the Hannum and PhenoAge clocks (Pearson r > 0.8). Other correlations were in the range of r = 0.4-0.5. The full results are shown in Figure 1. We developed scatterplots to demonstrate the association between chronological age and epigenetic age using the 1st and 2nd generation clocks (Figure 2) and the pace of aging (Figure 3).

Our primary analysis tested the hypothesis that seeking social support would protect against accelerated biological aging resulting from experiences of discrimination. We modeled each epigenetic clock as a function of discrimination experiences, seeking social support, and their interaction, as well as a set of covariates (age, body mass index, smoking status, blood pressure, and number and age of children) using linear regression. Hypothesis testing focused on the coefficients for discrimination (reflecting the association of discrimination with biological aging in the absence of seeking social support), seeking social support (reflecting the association of social support with biological aging), and the interaction term (reflecting the moderation of the discrimination association by seeking social support).

**Figure 4:**
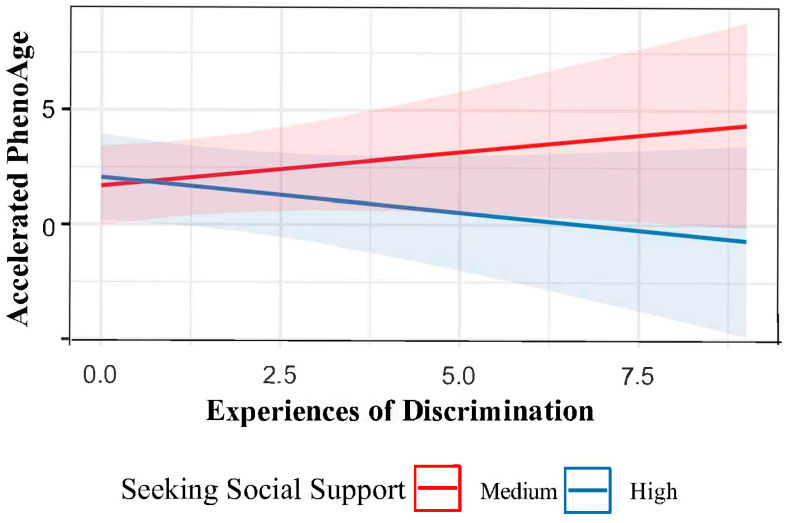
Interaction plot of maternal discrimination and seeking social support on PhenoAge acceleration. The figure shows the predicted interaction effect of seeking social support (Medium, red; High, blue) on the effect of experiences of discrimination on DNAm PhenoAge, a measure of biological aging, in *n* = 234 African American women participating in the InterGEN study.

The results of our primary analysis are shown in Table 2. We found evidence consistent with our hypothesis in the case of the PhenoAge clock (mean PhenoAge: 64.6 years, SD: 10.4), but not the other clocks. A greater number of experiences of discrimination were associated with older PhenoAge (b = 0.26, 95% Confidence Interval (CI) = 0.02–0.50, *p* = 0.03). However, social-support seeking mitigated this risk; at the highest levels of social support, no adverse consequences of discrimination were observed (interaction b = −0.01, 95% CI = −0.02–−0.00, *p* = 0.03) (Figure 4). The findings of the interaction term experiences of discrimination and social support were null for the other second- and third-generation epigenetic clocks (GrimAge: b = −0.00, 95% CI = −0.01–0.01, *p* = 0.8; DunedinPACE: b = −0.00, 95% CI = −0.01–0.01, *p* = 0.8) as well as first-generation epigenetic clocks (Horvath1: b = −0.00, 95% CI = −0.02–0.01, *p* = 0.4; Horvath2: b = −0.00, 95% CI = −0.01–0.01, *p* = 0.5; Hannum: b = −0.01, 95% CI = −0.02–0.00, *p* = 0.8). Statistically significant covariates included smoking (estimate: 0.98, 95% CI = 0.68–1.28, *p* < 0.001) and number of children (estimate: 0.12, 95% CI = 0.03–0.21, *p* = 0.01) in the GrimAge model and body mass index (estimate: 0.04; 95% CI = 0.02–0.06; *p* < 0.001) in the DunedinPACE model (Supplemental Table S1).

We also fitted simple models of the associations of discrimination and social support with biological aging. Results were null for all clocks tested in these models. Table 3 presents the association of discrimination with biological aging. Findings on the association between discrimination and biological aging were null for each epigenetic clock after adjusting for covariates (PhenoAge: b = 0.00, 95% CI = −0.06–0.07, *p* = 0.92, GrimAge: b = 0.00, 95% CI = −0.06–0.07, *p* = 0.95; DunedinPACE: b = −0.03, 95% CI = −0.09–0.04, *p* = 0.45; Horvath1: b = −0.01, 95% CI = −0.08–0.06, *p* = 0.85; Horvath2: b = −0.03, 95% CI = −0.10–0.04, *p* = 0.46; Hannum: b = −0.01, 95% CI = −0.08–0.06, *p* = 0.77). Statistically significant covariates included smoking (estimate: 0.98, 95% CI = 0.69–1.27, *p* < 0.001) and number of children (estimate: 0.12, 95% CI = 0.03–0.21, *p* = 0.01) in the GrimAge model and body mass index (estimate: 0.04; 95% CI = 0.02–0.06; *p* < 0.001) in the DunedinPACE model (Supplemental Table S2).

The associations of social support on biological aging are presented in Table 4. The findings on the association between social support and biological aging were null for each epigenetic clock after adjusting for covariates (PhenoAge: b = −0.01, 95% CI = −0.03–0.01, *p* = 0.41; GrimAge: b = −0.00, 95% CI = −0.02–0.02, *p* = 0.79; DunedinPACE: b = 0.00, 95% CI = −0.02–0.02, *p* = 0.79; Horvath1: b = −0.00, 95% CI = −0.02–0.02, *p* = 0.86; Horvath2: b = −0.00, 95% CI = −0.02–0.02, *p* = 0.88; Hannum: b = −0.01, 95% CI = −0.03–0.01, *p* = 0.19). Statistically significant covariates included smoking (estimate: 0.98, 95% CI = 0.69–1.28, *p* < 0.001) and number of children (estimate: 0.12, 95% CI = 0.03–0.21, *p* = 0.01) in the GrimAge model and body mass index (estimate: 0.04; 95% CI = 0.02–0.06; *p* < 0.001) in the DunedinPACE model (Supplemental Table S3).

Table 5 presents the model with the effects of both discrimination and seeking social support on biological aging. The findings on the association between discrimination (PhenoAge: b = 0.00, 95% CI = −0.06–0.07, *p* = 0.89; GrimAge: b = 0.00, 95% CI = −0.06–0.07, *p* = 0.94; DunedinPACE: b = −0.03, 95% CI = −0.09–0.04, *p* = 0.45; Horvath1: b = −0.01, 95% CI = −0.08–0.06, *p* = 0.85; Horvath2: b = −0.03, 95% CI = −0.10–0.04, *p* = 0.46; Hannum: b = −0.01, 95% CI = −0.08–0.06, *p* = 0.81) and social support (PhenoAge: b = −0.01, 95% CI = −0.03–0.01, *p* = 0.41; GrimAge: b = −0.00, 95% CI = −0.02–0.02, *p* = 0.78; DunedinPACE: b = 0.00, 95% CI = −0.02–0.02, *p* = 0.76; Horvath1: b = −0.00, 95% CI = −0.02–0.02, *p* = 0.87; Horvath2: b = 0.00, 95% CI = −0.02–0.02, *p* = 0.85; Hannum: b = −0.01, 95% CI = −0.03–0.01, *p* = 0.19) with biological aging were null for each epigenetic clock after adjusting for covariates. Statistically significant covariates included smoking (estimate: 0.98, 95% CI = 0.69–1.28, *p* < 0.001) and number of children (estimate: 0.12, 95% CI = 0.03–0.21, *p* = 0.01) in the GrimAge model and body mass index (estimate: 0.04; 95% CI = 0.02–0.06; *p* < 0.001) in the DunedinPACE model (Supplemental Table S4).

## 3. Discussion

We examined the associations between racial discrimination, coping, and biological aging measured using epigenetic clocks in this secondary analysis of data from a sample of young African American mothers. We hypothesized that racial discrimination experiences would be associated with older biological age and a faster pace of aging and that this association would be attenuated by the adaptive coping strategy of seeking social support. We found some support for this hypothesis in our analysis of the PhenoAge epigenetic clock. In the PhenoAge model, mothers who did not seek social support and experienced racial discrimination showed signs of accelerated biological aging, as indicated by an older PhenoAge. However, mothers who actively sought social support did not exhibit these adverse effects, even with a greater number of reported racial discrimination experiences. This suggests that seeking social support may protect against discrimination experiences on biological aging. However, for the other epigenetic clocks, the results did not support an effect of discrimination or moderation of it by social support. To our knowledge, this is the first study to explore the moderating effects of coping on the association between racial discrimination experiences and DNAm age acceleration among a sample of young African American mothers.

Our findings contribute to the existing literature on the association between psychosocial stressors and accelerated biological aging [61,63,64], with a particular focus on racial discrimination among young African American mothers. We built on our previous work in which we found seeking social support moderated the association between racial discrimination and blood pressure among this cohort [41]. In addition to experiencing racial discrimination, African American women have also reported discrimination based on gender [10,65,66] and sexuality [65,67]. To expand on these findings, future research should explore the longitudinal impacts of multiple forms of discrimination reported by African American mothers on biological aging while also considering specific reasons discrimination may be experienced and the coping strategies used to manage the experience. Discrimination as either an acute or chronic stressor may influence biological aging through different mechanisms; it is necessary to identify how these different types of discrimination may influence biological aging over time. Prior literature has assessed associations between different types of discrimination and biological aging over time [68,69,70,71,72], but few studies have focused on African American women [44,73]. Given there is some literature identifying an association between at least one form of discrimination and biological aging, future studies should also move toward models that represent the social dynamics through which one or combined forms of discrimination impact health. This study found some promise in that approach, but the inconsistent results suggest that larger samples may be needed to generate robust estimates of how risk and protective factors combine to influence biological aging in African American women.

In addition to exploring psychosocial factors affecting DNAm, additional literature on biological factors on epigenetic aging among young African American women is also needed. Prior literature has shown that biological factors, including cardiovascular [74,75] and metabolic risk factors [76], may influence epigenetic aging, but there is a gap in literature exploring such associations in young African American women. Additionally, social support or other coping strategies may modulate these associations and should be explored further.

We found that greater levels of seeking-social-support coping may, at least partially, buffer the effects of racial discrimination experiences on biological aging. African American women employ diverse strategies to cope with both acute and chronic stress. One framework which analyzes stress and coping among African American women is the Superwoman Schema [77]. Schemas are internal mental models that can develop from past experiences and consist of core beliefs, emotions, and memories; they shape how an individual interprets and responds to the social world [78]. The Superwoman Schema is characterized by traits that include strength, emotional suppression, resistance to vulnerability and dependence, the determination to succeed, and an obligation to help others [77]. While the characteristics of the Superwoman Schema can foster resilience and self-efficacy, particularly in the face of racial discrimination and chronic stress [79], they may also carry significant personal costs, including challenges with emotion regulation and increased psychological distress [77,80]. Over time, these cumulative stressors can contribute to elevated physical health risks, including cardiovascular disease [81,82], cancer [83], and persistent risky behaviors such as cigarette smoking [84,85]. Stress and coping strategies may indirectly influence biological aging through mechanisms such as DNAm, yet more research explaining how coping may influence healthy aging is needed. We studied only the social support coping strategy. Other adaptive (e.g., religion/prayer, problem solving) and maladaptive (e.g., avoidance, distancing) strategies may further explain our results. Future research should further explore these and additional coping strategies employed by African American women and mothers to better understand both protective and risk factors influencing healthy aging.

The results were inconsistent across the panel of epigenetic clocks tested in our study. We observed findings consistent with our hypothesis for the PhenoAge clock, but not for the other clocks. This is somewhat surprising given past studies observing evidence that both the GrimAge and DunedinPACE clocks indicate an older biological age and a faster pace of aging in African Americans and people exposed to stress and discrimination [20,24,61,71,86,87]. However, inconsistent results across clocks are common in the literature. These inconsistencies may reflect differences in the biological information captured by the different clocks as well as imprecision in the clock measurements [88,89] and in statistical estimates. Our analysis relied on versions of first- and second-generation epigenetic clocks computed using the PhenoAge clock method, which substantially improves precision in measurement [88]. The mixed results in our study thus likely reflect either substantive differences in the clocks or the limited precision of our association estimates owing to the relatively small size of our sample. Replication in larger samples is needed.

Recent work has suggested that epigenetic aging may partially reflect the accumulation of somatic mutations, particularly Cytosine-to-Thiamine transitions at methylated cytosines, which may be irreversible markers of epigenetic aging [90]. While this perspective complicates the interpretation of epigenetic clocks, it does not preclude the influence of psychosocial stressors or protective factors like social support on the more dynamic components of the methylome. Our findings highlight how coping strategies may buffer stress-related epigenetic aging, even within the broader context of mutation-linked biological change.

### Limitations

This study contributes to addressing a gap in the literature; however, several limitations should be considered when interpreting the findings. First, the cross-sectional design limits the ability to establish causality, making it difficult to determine the directionality of the relationship between racial discrimination and epigenetic aging; additionally, our findings cannot address the incidence of DNAm age acceleration [91]. Second, the sample, consisting exclusively of African American mothers from a single region of the United States, reduces the generalizability of the results to other populations, including African Americans in other regions or individuals of diverse racial and ethnic backgrounds. Third, other common coping strategies used among African American women, such as religious practices, were not accounted for in our study, and may partially explain the observed relationships [92]. Fourth, some researchers have suggested that the existing panels of epigenetic clocks, which were developed from an analysis of samples that were mostly or entirely White, may be less precise in measuring biological age when implemented in non-White samples [93]. Fifth, most of the clocks we examined were trained using DNA exclusively derived from whole blood. DNA in saliva samples comes from a combination of leukocytes and buccal epithelial cells. It remains uncertain whether clock values computed from saliva DNAm have comparable precision to clock values computed from whole-blood DNAm. The replication of results in whole-blood data would strengthen confidence in the findings. Additional research in a more diverse sample is needed to confirm our findings. Finally, discrimination stress and social support, while well-documented to have important effects on maternal health, only affected one of the six epigenetic clocks we examined, even without any statistical corrections for multiple comparisons. This suggests that epigenetic clocks may not be particularly sensitive to these kinds of social exposure. Despite these limitations, there remains a scarcity of datasets containing both genomic data and measures of racial discrimination experiences and coping strategies among African American women. The Intergenerational Impact of Genetic and Psychological Factors on Blood Pressure (InterGEN) cohort data provide a valuable opportunity to examine epigenetic aging and racial discrimination experiences in an understudied population facing disproportionate health risks. Future research addressing these limitations while prioritizing diverse and representative sampling of African Americans will be essential to advancing knowledge in this area.

## 4. Materials and Methods

### 4.1. Study Design and Sample

In this secondary analysis of data from the Intergenerational Impact of Genetic and Psychological Factors on Blood Pressure (InterGEN) study, we examined whether (1) racial discrimination influenced DNA methylation (DNAm) age acceleration and (2) seeking social support moderated this association in a sample of young African American mothers. The InterGEN Study [94,95] was a longitudinal cohort study (2014–2019) of 250 African American/Black mother/child dyads (n = 500) from socioeconomically disadvantaged communities in Connecticut, United States. The parent study aimed to examine the gene–environment interactions on blood pressure in African American women and their young children over time. Research team members recruited participants from early childhood education centers and through community outreach events in Connecticut. Eligible participants were (1) at least 21 years old, (2) self-identified as African American or Black, (3) spoke English, (4) had no mental illness which could interfere with psychological measures, and (5) enrolled with a biological child aged 3–5 years old. Written informed consent was obtained from all interested study participants during the enrollment interview. Research team members conducted interviews at four time points: T1 (baseline), T2 (6 months), T3 (12 months), and T4 (18 months). The Yale University Institutional Review Board approved the study (#1311012986).

Research team members collected demographic, clinical (height/weight, blood pressure, saliva for DNA analysis), and psychological measures (race-related trauma) data at the baseline visit. Audio–Computer-Assisted Self-Interview (ACASI) software (version 16) was used to collect psychological measures and demographic information (including mother’s self-reported smoking status). Full study procedures are available elsewhere [94,95].

### 4.2. Instruments and Measures

#### 4.2.1. Perceived Discrimination

Experiences of Discrimination Scale. The Experiences of Discrimination (EOD) scale measures self-reported experiences of racial discrimination in adults of all races/ethnicities from working backgrounds [13,96]. The EOD Situation subscale includes a nine-item questionnaire which asks respondents about different situations where they have experienced discrimination due to race, ethnicity, or skin color. Example settings include work, school, when obtaining housing, and from police or in the court system. EOD Situation scores range from 0 to 9. This sample has been validated in samples of adults of various races/ethnicities [32,97].

Coping. Seeking-social-support coping strategies were captured using the seeking social support subscale from the Coping Strategies Indicator (CSI) [98]. The CSI asks participants to recall one stressful event within the last six months and answer questions regarding their response. Items were scored using a Likert scale of 1 (not at all) to 3 (a lot). Scores for this subscale range from 11 to 33, with higher scores indicating greater use of seeking social support as a coping strategy.

#### 4.2.2. DNA Data Collection

Research team members collected saliva samples for DNA methylation analyses at baseline. The method of choice for collection and extraction of DNA was saliva because of the noninvasive nature of the collection, unlike blood, which would require a vein puncture. Oragene 500 Format tubes (DNA Genotek inc. Stittsville, ON Canada) were used to collect saliva samples. The OG-500 collection tube allowed for the extraction of approximately 110 μg of DNA [95,99]. Participants were asked to refrain from eating, drinking, smoking, or chewing gum for at least 30 min before saliva sample collection. Participants spit into the collection tube until the saliva reached the 2-milliliters fill line. Participants were given a clear sweetened lollipop if they were unable to produce enough saliva to reach the fill line. Barcodes were placed on each participants’ sample for tracking purposes. Samples were stored at 4 °C until DNA extraction and analysis was completed. The standard protocol for DNA extraction and purification was conducted as indicated in the standard operating procedures guidelines using ReliaPrep kits (Promega Corporation. Madison, WI. USA) and the Illumina Infinium Methylation EPIC (850 K) BeadChip (Illumina Inc. San Diego, CA, USA.) to analyze epigenome-wide DNA methylation [100,101]. This EPIC BeadChip directly quantified DNA methylation in CpG dinucleotides [95]. Hybridization was performed on a per-sample basis. These BeadChip arrays are well annotated for CpG island and non-CpG island promoters, shore regions, coding regions, repetitive elements, miRNA promoter regions, FANTOM5 enhancers, ENCODE open chromatin and enhancers, and DNase hypersensitivity sites and include 91.1% of the loci from the HumanMethylation450 BeadChip (Illumina Inc. San Diego, CA, USA) [95]. DNA methylation was determined at each CpG site on the 850 K array by the fluorescent signals from the methylated (M) and unmethylated (U) probes specific for each site. This covered approximately 99% of all RefSeq genes and 96% of CpG islands [95,100,101]. We used methylation-specific polymerase chain reaction [102] and bisulfite sequencing [103] to confirm DNA methylation [95].

### 4.3. DNA Methylation Preprocessing and Epigenetic Clock Calculation

We conducted the preprocessing and normalization of DNA methylation data in R (v 4.4.1) on *n* = 512 samples, comprising these women and their offspring, which included 14 replicates for mothers and 2 replicates for children. We used minfi (v1.5.0) [104] and ewastools packages (v 1.7.2) [88]. We removed samples with the following criteria: samples with a methylated or unmethylated signal intensity < 10 (n = 8); samples with a bisulfite conversion efficiency < 80% (*n* = 2); samples with average detection *p*-values < 0.05 (*n* = 2); samples with a non-polymorphic red signal intensity < 5 (*n* = 1); and samples where predicted sex differed from reported sex at birth (*n* = 12). Several samples failed more than one measure, resulting in the removal of n = 18 unique samples. A large number of samples (*n* = 160) belonging to one technical batch failed hybridization ratio tests but performed well on other quality metrics and were retained. In total, *n* = 248 samples belonging to mothers participating in this study passed quality control.

We performed normalization using the preprocessNoob function in the minfi package (v1.50.0). DNAmGrimAge [53] was calculated using the methods described by Higgins-Chen et al. [62]. We estimated epithelial and immune cell composition in saliva using the estimateIC function in ewastools (v 1.7.2) [88].

#### DNAm Age Estimation

Epigenetic clocks were calculated on Noob-normalized data using the methods described by Higgins-Chen et al. [62]. DunedinPACE was derived as described by Belsky et al. [55]. The epithelial and immune cell composition in saliva was estimated using the estimateIC function in ewastools (v 1.7.2) [88]. Prior to analysis, all epigenetic clock estimates were residualized (taking the residual from a regression of the clocks) on age and percent epithelial cell composition and scaled to a mean = 0, SD = 1.

### 4.4. Statistical Analysis

We built separate linear regression models to assess the interaction effects between the EOD Situation and the CSI scale on each accelerated methylation age, respectively. We reran the model without the interaction between EOD Situation and coping strategy if the interaction effect was not significant. We controlled for mothers’ age, mothers’ body mass index (kg/m^2^), mothers’ smoking status (Yes/No), mothers’ systolic and diastolic blood pressures, the number of biological and adopted children in the family, child’s sex-assigned-at-birth, child’s age, proportion of epithelial cells, and sample batch as covariates in each model. All analyses were performed in R version 4.4.1. Test significance was set at a 0.05 level.

## 5. Conclusions

This study aimed to address the association between racial discrimination, coping, and DNAm age acceleration in a sample of young African American mothers. We found that seeking social support coping was a significant moderator of the association between racial discrimination and DNAm age acceleration using the PhenoAge epigenetic clock. While this study had limitations, this secondary data analysis adds to the literature addressing racially charged events and healthy aging among young African American mothers. Our findings reinforce the need for research addressing associations between social determinants of health and healthy aging [105]. Longitudinal studies with larger, more diverse samples are warranted to confirm and expand upon these findings.

## Figures and Tables

**Table 1:** Baseline Profile.

Sample Characteristics (*n* = 234)	Mean (SD) *n* (%)	Median [Min, Max]
Maternal Age	31.9 (5.80)	31.3 [21.0, 46.8]
Body Mass Index	29.6 (8.13)	28.6 [13.7, 59.0]
Systolic Blood Pressure	14 (13.5)	113 [81.3, 163]
Diastolic Blood Pressure	73.0 (10.6)	72.0 [50.0, 110]
Smoking Status		
No	181 (77.4%)	
Yes	53 (22.6%)	
Number of children	2.56 (1.54)	2.00 [1, 10.0]
Child Age	4.14 (0.78)	4.10 [3.00, 5.90]
Experiences of Discrimination, Situation	1.45 (1.93)	1.00 [0, 9.00]
Seeking Social Support	21.7 (7.2)	20.8 [11, 33]

**Table 2:** Adjusted moderation analyses of discrimination, coping, and epigenetic aging. All epigenetic age measures were residualized on age and percent of epithelial cell composition and scaled to mean = 0, SD = 1.

	PCHorvath1		PCHorvath2		PCHannum		PCPhenoAge		PCGrimAge		DunedinPACE	
Predictors	Estimates	*p*	Estimates	*p*	Estimates	*p*	Estimates	*p*	Estimates	*p*	Estimates	*p*
(Intercept)	−1.18 (−2.76–0.40)	0.1	−1.57 (−3.15–0.00)	0.5	−0.46 (−2.01–1.09)	0.6	−1.69 (−3.21–−0.17)	0.03	−1.39 (−2.83–0.06)	0.6	−1.81 (−3.31–−0.31)	0.02
Experiences of Discrimination	0.11 (−0.14–0.36)	0.4	0.05 (−0.20–0.31)	0.7	0.21 (−0.04–0.45)	0.1	0.26 (0.02–0.50)	**0.03**	0.03 (−0.20–0.26)	0.8	0.00 (−0.24–0.24)	1.0
Social Support	0.00 (−0.02–0.03)	0.7	0.01 (−0.02–0.03)	0.6	−0.00 (−0.03–0.02)	0.9	0.01 (−0.02–0.03)	0.6	−0.00 (−0.02–0.02)	0.9	0.00 (−0.02–0.03)	0.7
Experiences of Discrimination × Social Support	−0.00 (−0.02–0.01)	0.4	−0.00 (−0.01–0.01)	0.5	−0.01 (−0.02–0.00)	0.8	−0.01 (−0.02–−0.00)	**0.03**	−0.00 (−0.01–0.01)	0.8	−0.00 (−0.01–0.01)	0.8
Observations	234		234	234		234		234			234	
R^2^/R^2^ adjusted	0.046/−0.011		0.049/−0.007	0.079/0.024		0.115/0.063		0.213/0.167			0.149/0.099	

bold represents *p* value <0.05.

**Table 3:** Adjusted analyses of discrimination on epigenetic aging. All epigenetic age measures were residualized on age and percent of epithelial cell composition and scaled to mean = 0, SD = 1.

	PCHorvath1		PCHorvath2		PCHannum		PCPhenoAge		PCGrimAge		DunedinPACE	
Predictors	Estimates	*p*	Estimates	*p*	Estimates	*p*	Estimates	*p*	Estimates	*p*	Estimates	*p*
(Intercept)	−1.14 (−2.59–0.31)	0.12	−1.46 (−2.90–−0.01)	0.05	−0.68 (−2.12–0.76)	0.35	−1.73 (−3.15–−0.32)	0.02	−1.44 (−2.76–−0.11)	0.03	−1.71 (−3.08–−0.33)	0.02
Experiences of Discrimination	−0.01 (−0.08–0.06)	0.85	−0.03 (−0.10–0.04)	0.46	−0.01 (−0.08–0.06)	0.77	0.00 (−0.06–0.07)	0.92	0.00 (−0.06–0.07)	0.95	−0.03 (−0.09–0.04)	0.45
Observations	234		234		234		234		234		234	
R^2^/R^2^ adjusted	0.042/−0.006		0.047/0.000		0.058/0.011		0.093/0.049		0.213/0.174		0.148/0.106	

**Table 4:** Adjusted analyses of seeking social support and epigenetic aging. All epigenetic age measures were residualized on age and percent of epithelial cell composition and scaled to mean = 0, SD = 1.

	PCHorvath1		PCHorvath2		PCHannum		PCPhenoAge		PCGrimAge		DunedinPACE	
Predictors	Estimates	*p*	Estimates	*p*	Estimates	*p*	Estimates	*p*	Estimates	*p*	Estimates	*p*
(Intercept)	−1.10 (−2.66–0.46)	0.17	−1.54 (−3.10–0.02)	0.05	−0.30 (−1.85–1.24)	0.70	−1.49 (−3.01–0.03)	0.06	−1.36 (−2.79–0.06)	0.06	−1.82 (−3.30–−0.33)	0.02
Seeking Social Support	−0.00 (−0.02–0.02)	0.86	0.00 (−0.02–0.02)	0.88	−0.01 (−0.03–0.01)	0.19	−0.01 (−0.03–0.01)	0.41	−0.00 (−0.02–0.02)	0.79	0.00 (−0.02–0.02)	0.79
Observations	234		234		234		234		234		234	
R^2^/R^2^ adjusted	0.042/−0.006		0.045/−0.002		0.065/0.019		0.096/0.051		0.213/0.174		0.147/0.104	

**Table 5:** Adjusted analyses of discrimination, coping, and epigenetic aging. All epigenetic age measures were residualized on age and percent epithelial cell composition and scaled to mean = 0, SD = 1.

	PCHorvath1		PCHorvath2		PCHannum		PCPhenoAge		PCGrimAge		DunedinPACE	
Predictors	Estimates	*p*	Estimates	*p*	Estimates	*p*	Estimates	*p*	Estimates	*p*	Estimates	*p*
(Intercept)	−1.09 (−2.66–0.48)	0.17	−1.51 (−3.07–0.05)	0.06	−0.29 (−1.84–1.26)	0.71	−1.49 (−3.02–0.03)	0.55	−1.36 (−2.79–0.07)	0.06	−1.79 (−3.28–−0.30)	0.02
Experiences of Discrimination	−0.01 (−0.08–0.06)	0.85	−0.03 (−0.10–0.04)	0.46	−0.01 (−0.08–0.06)	0.81	0.00 (−0.06–0.07)	0.89	0.00 (−0.06–0.07)	0.94	−0.03 (−0.09–0.04)	0.45
Seeking Social Support	−0.00 (−0.02–0.02)	0.87	0.00 (−0.02–0.02)	0.85	−0.01 (−0.03–0.01)	0.19	−0.01 (−0.03–0.01)	0.41	−0.00 (−0.02–0.02)	0.78	0.00 (−0.02–0.02)	0.76
Observations	234		234		234		234		234		234	
R^2^/R^2^ adjusted	0.042/−0.010		0.047/−0.004		0.065/0.015		0.096/0.047		0.213/0.170		0.149/0.103	

## Data Availability

The data that support the findings of this study are available from the final author upon reasonable request.

## Supplementary Materials

The following supporting information can be downloaded at: https://www.mdpi.com/article/10.3390/epigenomes9020014/s1, Table S1: Adjusted Moderation Analyses of Discrimination, Coping, and Epigenetic Aging. All epigenetic age measures were residualized on age and percent epithelial cell composition and scaled to mean = 0, SD = 1; Table S2: Adjusted Analyses of Discrimination on Epigenetic Aging. All epigenetic age measures were residualized on age and percent epithelial cell composition and scaled to mean = 0, SD = 1; Table S3: Adjusted Analyses of Seeking Social Support and Epigenetic Aging. All epigenetic age measures were residualized on age and percent epithelial cell composition and scaled to mean = 0, SD = 1; Table S4: Adjusted Analyses of Discrimination, Coping, and Epigenetic Aging. All epigenetic age measures were residualized on age and percent epithelial cell composition and scaled to mean=0, SD=1.

## Abbreviations

The following abbreviations are used in this manuscript:

ACASI	Audio–Computer-Assisted Self-Interview
CSI	Coping Strategies Indicator
DNAm	DNA methylation
EOD	Experiences of Discrimination
InterGEN	Intergenerational Impact of Genetic and Psychological Factors on Blood Pressure
IRB	Institutional Review Board
SD	Standard deviation
