# Supplementary material for: Discrimination, Coping, and DNAm Accelerated Aging Among African American Mothers of the InterGEN Study
Source: Epigenomes. 2025 May 4;9(2):14. doi: 10.3390/epigenomes9020014 (PMC12101303; doi:10.3390/epigenomes9020014)
Supplement: Supplementary file 1 [file epigenomes-09-00014-s001.zip › epigenomes-3536896-supplementary.pdf]

Supplement Tables

Table S1. Adjusted Moderation Analyses of Discrimination, Coping, and Epigenetic Aging. All epigenetic age measures were residualized on age and percent epithelial cell composition and scaled to mean=0, SD=1.

	**PCHorvath1**		**PCHorvath2**		**PCHannum**		**PCPhenoAge**		**PCGrimAge**		**DunedinPACE**	
Predictors	*Estimates*	*p*	*Estimates*	*p*	*Estimates*	*p*	*Estimates*	*p*	*Estimates*	*p*	*Estimates*	*p*
(Intercept)	-1.18 (-2.76 – 0.40)	0.1	-1.57 (-3.15 – 0.00)	0.5	-0.46 (-2.01 – 1.09)	0.6	-1.69 (-3.21 – -0.17)	0.03	-1.39 (-2.83 – 0.06)	0.6	-1.81 (-3.31 – -0.31)	0.02
Maternal Age	-0.01 (-0.04 – 0.01)	0.3	-0.01 (-0.04 – 0.02)	0.5	0.00 (-0.03 – 0.03)	1.0	0.00 (-0.03 – 0.03)	1.0	-0.00 (-0.03 – 0.02)	0.8	-0.01 (-0.03 – 0.02)	0.6
Body Mass Index	0.01 (-0.01 – 0.03)	0.3	0.01 (-0.01 – 0.03)	0.2	-0.00 (-0.02 – 0.01)	0.6	0.01 (-0.00 – 0.03)	0.2	0.00 (-0.01 – 0.02)	0.6	0.04 (0.02 – 0.06)	**<0.001**
Systolic blood pressure	0.01 (-0.01 – 0.02)	0.5	0.01 (-0.01 – 0.02)	0.3	-0.00 (-0.02 – 0.01)	0.7	0.01 (-0.01 – 0.02)	0.3	0.01 (-0.00 – 0.03)	0.1	0.01 (-0.01 – 0.02)	0.5
Diastolic blood pressure	0.00 (-0.02 – 0.02)	0.7	0.00 (-0.02 – 0.02)	0.8	0.00 (-0.02 – 0.02)	0.9	-0.01 (-0.03 – 0.01)	0.4	-0.01 (-0.03 – 0.01)	0.3	-0.01 (-0.02 – 0.01)	0.5
Smoker (Yes)	-0.06 (-0.38 – 0.27)	0.7	0.09 (-0.23 – 0.42)	0.6	0.00 (-0.32 – 0.32)	1.0	0.06 (-0.25 – 0.38)	0.7	0.98 (0.68 – 1.28)	**<0.001**	0.27 (-0.03 – 0.58)	0.1
Number of Children	0.03 (-0.06 – 0.12)	0.5	0.04 (-0.05 – 0.13)	0.4	0.07 (-0.02 – 0.16)	0.1	0.08 (-0.02 – 0.17)	0.1	0.12 (0.03 – 0.21)	**0.01**	0.06 (-0.03 – 0.14)	0.2
Child sex	-0.02 (-0.30 – 0.25)	0.9	0.00 (-0.27 – 0.28)	1.0	-0.07 (-0.34 – 0.20)	0.6	0.03 (-0.23 – 0.30)	0.8	-0.02 (-0.27 – 0.23)	0.9	0.03 (-0.23 – 0.29)	0.8
Child age	0.09 (-0.09 – 0.27)	0.3	0.05 (-0.13 – 0.22)	0.6	0.14 (-0.04 – 0.31)	0.1	0.09 (-0.08 – 0.26)	0.3	-0.02 (-0.18 – 0.14)	0.8	0.05 (-0.12 – 0.22)	0.5
Experiences of Discrimination	0.11 (-0.14 – 0.36)	0.4	0.05 (-0.20 – 0.31)	0.7	0.21 (-0.04 – 0.45)	0.1	0.26 (0.02 – 0.50)	**0.03**	0.03 (-0.20 – 0.26)	0.8	0.00 (-0.24 – 0.24)	1.0
Social Support	0.00 (-0.02 – 0.03)	0.7	0.01 (-0.02 – 0.03)	0.6	-0.00 (-0.03 – 0.02)	0.9	0.01 (-0.02 – 0.03)	0.6	-0.00 (-0.02 – 0.02)	0.9	0.00 (-0.02 – 0.03)	0.7
Experiences of Discrimination X Social Support	-0.00 (-0.02 – 0.01)	0.4	-0.00 (-0.01 – 0.01)	0.5	-0.01 (-0.02 – 0.00)	0.8	-0.01 (-0.02 – -0.00)	**0.03**	-0.00 (-0.01 – 0.01)	0.8	-0.00 (-0.01 – 0.01)	0.8

Observations	234	234	234	234	234	234
R^2 / R^2 adjusted	0.046 / -0.011	0.049 / -0.007	0.079 / 0.024	0.115 / 0.063	0.213 / 0.167	0.149 / 0.099

Table S2. Adjusted Analyses of Discrimination on Epigenetic Aging. All epigenetic age measures were residualized on age and percent epithelial cell composition and scaled to mean=0, SD=1.

	**PCHorvath1**		**PCHorvath2**		**PCHannum**		**PCPhenoAge**		**PCGrimAge**		**DunedinPACE**	
Predictors	*Estimates*	*p*	*Estimates*	*p*	*Estimates*	*p*	*Estimates*	*p*	*Estimates*	*p*	*Estimates*	*p*
(Intercept)	-1.14 (-2.59 – 0.31)	0.12	-1.46 (-2.90 – -0.01)	0.05	-0.68 (-2.12 – 0.76)	0.35	-1.73 (-3.15 – -0.32)	0.02	-1.44 (-2.76 – -0.11)	0.03	-1.71 (-3.08 – -0.33)	0.02
Maternal age	-0.01 (-0.04 – 0.01)	0.32	-0.01 (-0.04 – 0.02)	0.45	0.00 (-0.02 – 0.03)	0.88	0.00 (-0.03 – 0.03)	0.93	-0.00 (-0.03 – 0.02)	0.86	-0.01 (-0.03 – 0.02)	0.59
Body mass index	0.01 (-0.01 – 0.03)	0.31	0.01 (-0.01 – 0.03)	0.23	-0.00 (-0.02 – 0.01)	0.59	0.01 (-0.01 – 0.03)	0.20	0.00 (-0.01 – 0.02)	0.65	0.04 (0.02 – 0.06)	**<0.001**
Systolic blood pressure	0.01 (-0.01 – 0.02)	0.44	0.01 (-0.01 – 0.02)	0.31	-0.00 (-0.02 – 0.01)	0.87	0.01 (-0.01 – 0.03)	0.24	0.01 (-0.00 – 0.03)	0.10	0.01 (-0.01 – 0.02)	0.47
Diastolic blood pressure	0.00 (-0.02 – 0.02)	0.68	0.00 (-0.02 – 0.02)	0.75	0.00 (-0.02 – 0.02)	0.95	-0.01 (-0.03 – 0.01)	0.38	-0.01 (-0.03 – 0.01)	0.34	-0.01 (-0.02 – 0.01)	0.55
Smoker (Yes)	-0.05 (-0.37 – 0.27)	0.76	0.10 (-0.22 – 0.42)	0.54	0.01 (-0.31 – 0.33)	0.97	0.08 (-0.24 – 0.39)	0.64	0.98 (0.69 – 1.27)	**<0.001**	0.28 (-0.03 – 0.58)	0.07
Number of children	0.03 (-0.06 – 0.12)	0.54	0.04 (-0.05 – 0.13)	0.39	0.06 (-0.03 – 0.16)	0.17	0.07 (-0.02 – 0.17)	0.11	0.12 (0.03 – 0.21)	**0.001**	0.06 (-0.03 – 0.15)	0.22
Child Sex	-0.04 (-0.31 – 0.24)	0.79	-0.00 (-0.28 – 0.27)	0.97	-0.10 (-0.37 – 0.17)	0.47	-0.00 (-0.27 – 0.27)	1.00	-0.03 (-0.28 – 0.22)	0.82	0.03 (-0.23 – 0.29)	0.83
Child age	0.09 (-	0.31	0.05 (-	0.61	0.14 (-	0.11	0.09 (-	0.28	-0.02 (-0.18 – 0.14)	0.82	0.05 (-0.12 – 0.22)	0.55

	0.08 – 0.27)		0.13 – 0.22)		0.03 – 0.32)		0.08 – 0.27)					
Experiences of Discrimination	-0.01 (-0.08 – 0.06)	0.85	-0.03 (-0.10 – 0.04)	0.46	-0.01 (-0.08 – 0.06)	0.77	0.00 (-0.06 – 0.07)	0.92	0.00 (-0.06 – 0.07)	0.95	-0.03 (-0.09 – 0.04)	0.45
Observations	234		234		234		234		234		234	
R^2 / R^2 adjusted	0.042 / -0.006		0.047 / 0.000		0.058 / 0.011		0.093 / 0.049		0.213 / 0.174		0.148 / 0.106	

Table S3. Adjusted Analyses of Seeking Social Support and Epigenetic Aging. All epigenetic age measures were residualized on age and percent epithelial cell composition and scaled to mean=0, SD=1.

	**PCHorvath1**		**PCHorvath2**		**PCHannum**		**PCPhenoAge**		**PCGrimAge**		**DunedinPACE**	
Predictors	*Estimates*	*p*	*Estimates*	*p*	*Estimates*	*p*	*Estimates*	*p*	*Estimates*	*p*	*Estimates*	*p*
(Intercept)	-1.10 (-2.66 – 0.46)	0.17	-1.54 (-3.10 – 0.02)	0.05	-0.30 (-1.85 – 1.24)	0.70	-1.49 (-3.01 – 0.03)	0.06	-1.36 (-2.79 – 0.06)	0.06	-1.82 (-3.30 – -0.33)	**0.02**
Maternal age	-0.01 (-0.04 – 0.01)	0.29	-0.01 (-0.04 – 0.02)	0.39	0.00 (-0.03 – 0.03)	0.97	0.00 (-0.03 – 0.03)	0.96	-0.00 (-0.03 – 0.02)	0.85	-0.01 (-0.03 – 0.02)	0.52
Body mass index	0.01 (-0.01 – 0.03)	0.32	0.01 (-0.01 – 0.03)	0.22	-0.01 (-0.02 – 0.01)	0.54	0.01 (-0.01 – 0.03)	0.22	0.00 (-0.01 – 0.02)	0.66	0.04 (0.02 – 0.06)	**<0.001**
Systolic blood pressure	0.01 (-0.01 – 0.02)	0.44	0.01 (-0.01 – 0.02)	0.30	-0.00 (-0.02 – 0.01)	0.85	0.01 (-0.01 – 0.03)	0.25	0.01 (-0.00 – 0.03)	0.10	0.01 (-0.01 – 0.02)	0.45
Diastolic blood pressure	0.00 (-0.02 – 0.02)	0.67	0.00 (-0.02 – 0.02)	0.71	0.00 (-0.02 – 0.02)	0.92	-0.01 (-0.03 – 0.01)	0.38	-0.01 (-0.03 – 0.01)	0.341	-0.01 (-0.02 – 0.01)	0.58
Smoker (Yes)	-0.05 (-0.37 – 0.27)	0.76	0.10 (-0.23 – 0.42)	0.56	0.02 (-0.30 – 0.33)	0.92	0.08 (-0.23 – 0.40)	0.61	0.98 (0.69 – 1.28)	**<0.001**	0.27 (-0.03 – 0.58)	0.08
Number of children	0.03 (-	0.53	0.04 (-	0.41	0.07 (-	0.14	0.08 (-	0.10	0.12 (0.04 – 0.21)	**0.01**	0.05 (-0.03 – 0.14)	0.23

	0.06 – 0.12)		0.05 – 0.13)		0.02 – 0.16)		0.01 – 0.17)					
Child sex	-0.04 (-0.31 – 0.24)	0.80	-0.00 (-0.27 – 0.27)	0.99	-0.09 (-0.36 – 0.18)	0.50	0.00 (-0.26 – 0.27)	0.99	-0.03 (-0.28 – 0.22)	0.83	0.03 (-0.23 – 0.29)	0.81
Child age	0.09 (-0.09 – 0.27)	0.31	0.04 (-0.13 – 0.22)	0.63	0.14 (-0.04 – 0.31)	0.12	0.09 (-0.08 – 0.26)	0.29	-0.02 (-0.18 – 0.14)	0.81	0.05 (-0.12 – 0.22)	0.57
Seeking Social Support	-0.00 (-0.02 – 0.02)	0.86	0.00 (-0.02 – 0.02)	0.88	-0.01 (-0.03 – 0.01)	0.19	-0.01 (-0.03 – 0.01)	0.41	-0.00 (-0.02 – 0.02)	0.79	0.00 (-0.02 – 0.02)	0.79
Observations	234		234		234		234		234		234	
R^2 / R^2 adjusted	0.042 / -0.006		0.045 / -0.002		0.065 / 0.019		0.096 / 0.051		0.213 / 0.174		0.147 / 0.104	

Table S4. Adjusted Analyses of Discrimination, Coping, and Epigenetic Aging. All epigenetic age measures were residualized on age and percent epithelial cell composition and scaled to mean=0, SD=1.

	**PCHorvath1**		**PCHorvath2**		**PCHannum**		**PCPhenoAge**		**PCGrimAge**		**DunedinPACE**	
Predictors	*Estimates*	*p*	*Estimates*	*p*	*Estimates*	*p*	*Estimates*	*p*	*Estimates*	*p*	*Estimates*	*p*
(Intercept)	-1.09 (-2.66 – 0.48)	0.17	-1.51 (-3.07 – 0.05)	0.06	-0.29 (-1.84 – 1.26)	0.71	-1.49 (-3.02 – 0.03)	0.55	-1.36 (-2.79 – 0.07)	0.06	-1.79 (-3.28 – -0.30)	**0.02**
Maternal age	-0.01 (-0.04 – 0.01)	0.31	-0.01 (-0.04 – 0.02)	0.46	0.00 (-0.03 – 0.03)	0.95	0.00 (-0.03 – 0.03)	0.97	-0.00 (-0.03 – 0.02)	0.85	-0.01 (-0.03 – 0.02)	0.61
Body mass index	0.01 (-0.01 – 0.03)	0.32	0.01 (-0.01 – 0.03)	0.22	-0.01 (-0.02 – 0.01)	0.54	0.01 (-0.01 – 0.03)	0.22	0.00 (-0.01 – 0.02)	0.66	0.04 (0.02 – 0.06)	**<0.001**
Systolic blood pressure	0.01 (-0.01 – 0.02)	0.44	0.01 (-0.01 – 0.02)	0.31	-0.00 (-0.02 – 0.01)	0.84	0.01 (-0.01 – 0.03)	0.25	0.01 (-0.00 – 0.03)	0.10	0.01 (-0.01 – 0.02)	0.47
Diastolic blood pressure	0.00 (-0.02 – 0.02)	0.68	0.00 (-0.02 – 0.02)	0.75	0.00 (-0.02 – 0.02)	0.93	-0.01 (-0.03 – 0.01)	0.39	-0.01 (-0.03 – 0.01)	0.35	-0.01 (-0.02 – 0.01)	0.55

Smoker (Yes)	-0.05 (-0.37 – 0.27)	0.76	0.10 (-0.22 – 0.42)	0.55	0.02 (-0.30 – 0.34)	0.92	0.08 (-0.23 – 0.40)	0.61	0.98 (0.69 – 1.28)	**<0.001**	0.28 (-0.03 – 0.58)	0.08
Number of children	0.03 (-0.06 – 0.12)	0.53	0.04 (-0.05 – 0.13)	0.40	0.07 (-0.02 – 0.16)	0.14	0.08 (-0.02 – 0.17)	0.10	0.12 (0.03 – 0.21)	**0.01**	0.06 (-0.03 – 0.14)	0.23
Child sex	-0.04 (-0.31 – 0.24)	0.79	-0.01 (-0.28 – 0.27)	0.97	-0.09 (-0.36 – 0.18)	0.50	0.00 (-0.26 – 0.27)	0.98	-0.03 (-0.28 – 0.22)	0.83	0.03 (-0.23 – 0.29)	0.83
Child age	0.09 (-0.09 – 0.27)	0.31	0.05 (-0.13 – 0.22)	0.60	0.14 (-0.04 – 0.31)	0.12	0.09 (-0.08 – 0.26)	0.29	-0.02 (-0.18 – 0.14)	0.81	0.05 (-0.12 – 0.22)	0.54
Experiences of Discrimination	-0.01 (-0.08 – 0.06)	0.85	-0.03 (-0.10 – 0.04)	0.46	-0.01 (-0.08 – 0.06)	0.81	0.00 (-0.06 – 0.07)	0.89	0.00 (-0.06 – 0.07)	0.94	-0.03 (-0.09 – 0.04)	0.45
Seeking Social Support	-0.00 (-0.02 – 0.02)	0.87	0.00 (-0.02 – 0.02)	0.85	-0.01 (-0.03 – 0.01)	0.19	-0.01 (-0.03 – 0.01)	0.41	-0.00 (-0.02 – 0.02)	0.78	0.00 (-0.02 – 0.02)	0.76
Observations	234		234		234		234		234		234	
R^2 / R^2 adjusted	0.042 / -0.010		0.047 / -0.004		0.065 / 0.015		0.096 / 0.047		0.213 / 0.170		0.149 / 0.103	
